# Accelerating the Front End of Medicine: Three Digital Use Cases and HCI Implications

**DOI:** 10.3390/healthcare10112176

**Published:** 2022-10-30

**Authors:** Matthias Klumpp, André Hanelt, Maike Greve, Lutz M. Kolbe, Schahin Tofangchi, Florian Böhrnsen, Jens Jakob, Sylvia Kaczmarek, Ingo Börsting, Christopher Ehmke, Helena Düsing, Christian Juhra

**Affiliations:** 1 Fraunhofer IML Dortmund, 44227 Dortmund, Germany; 2 Department of Business Administration, University of Göttingen, 37073 Göttingen, Germany; 3 Faculty of Economics and Management, University of Kassel, 34125 Kassel, Germany; 4 Soramitsu Co., Ltd., Tokyo 151-0051, Japan; 5 University Medical Center Göttingen, 37075 Göttingen, Germany; 6 Faculty of Business Administration and Economics, University of Duisburg-Essen, 45127 Essen, Germany; 7 University Medical Center of Münster, 48149 Münster, Germany

**Keywords:** digital health care, human-centered computing, emergency medicine, inpatient data management, cancer diagnosis, front end, artificial intelligence

## Abstract

Digital applications in health care are a concurrent research and management question, where implementation experiences are a core field of information systems research. It also contributes to fighting pandemic crises like COVID-19 because contactless information flow and speed of diagnostics are improved. This paper presents three digital application case studies from emergency medicine, administration management, and cancer diagnosis with AI support from the University Medical Centers of Münster and Göttingen in Germany. All cases highlight the potential of digitalization to increase speed and efficiency within the front end of medicine as the crucial phase before patient treatment starts. General challenges for health care project implementations and human-computer interaction (HCI) concepts in health care are derived and discussed, including the importance of specific processes together with user analysis and adaption. A derived concept for HCI includes the criteria speed, accuracy, modularity, and individuality to achieve sustainable improvements within the front end of medicine.

## 1. Introduction

Digitalization and digital applications are current topics in health care management research and practice (incl. eHealth and mHealth) [1,2,3,4,5,6,7,8]. This paper contributes to this discussion by presenting three real-life use cases of digital applications (i.e., desktop-based or mobile software solutions) aiming to improve health care at the University Medical Centers of Münster and Göttingen in Germany. These cases address what we define as the front end of medicine, that is, the processes and areas involved before actual patient treatment starts. The cases outline specific tasks within this front end in the emergency medicine, patient administration, and diagnosis fields. 

A comparison of the cases offers valuable insights regarding the shared challenges and features for digital applications in health care that address the human–computer interaction (HCI) domain. Health care systems are typically characterized by their need for a human-centered approach owing to the inclusion of high-priority questions, such as the timely provision of medical items, personnel, and resources to injured or ill people. The health technology sector has experienced rapid growth in recent years due to the high demand for digital services and products. Such services not only improve health care quality but also reduce cost and increase efficiency in hospital work processes [9]. 

The advantages of artificial intelligence (AI) implementation especially support these processes and provide the opportunity for automated data-driven analyses that increase efficiency and quality in medical treatment [10]. In this respect, HCI components play a vital role in guaranteeing the usability of such systems in complex work routines in hospitals [11]. Several stakeholders must be incorporated in their diverse requirements [12], including widespread technology interaction and digital systems communication [13]. Special focus can be directed toward the front end of medicine, including (1) all the emergency services outside the hospital, (2) all communications and data-handling processes dealing with inpatient information (i.e., from family doctors), and (3) all diagnostic endeavors before an actual therapy for a patient can be initiated. This front end constitutes a major playing field for digitalization because of three special characteristics. First, data processing may save time and resources and speed up actual patient treatment with a higher level of available resources. Second, scaling potentials may be huge as most institutions and actors work with similar processes (e.g., in the emergency medicine section), often fostered by common regulation frameworks, but currently work with different non-digital processes. Third, acquiring and training qualified personnel is becoming harder each day; therefore, human and non-digital solutions should be complemented by labor-saving systems sooner than later to maintain service and efficiency levels in hospitals, especially under severe pandemic conditions. In short, HCI as a basis for digital front-end medical processes is of high interest in the health care sector. 

Considerable research has been conducted to define HCI principles and develop evaluation models in information systems (IS). The beginnings of this research date back to the late 1970s, when computers from the back office came to the organizational front lines in the form of time-sharing systems and later in the form of office productivity tools on personal computers. The initial thematic focus was on the material characteristics of computers such as keyboard design or the ergonomic factors that influence user efficiency. Much of the later research, which ultimately distinguished HCI as a field in its own right, focused on how forms of computer interaction enabled or hindered user behavior based on the availability of designed computer functions. Hence the goal of HCI is to investigate the phenomenon of user interaction with technology and understand the implications of design on this interaction. The review of HCI-related publications shows a growing body of literature that uses models, principles, and rules to evaluate HCI elements such as usability, user experience, and aesthetics in different IS [14,15,16,17,18]. 

Altogether, some thoughts and resources have been invested in this important sector of HCI in general and within the health care and hospital management sub-field. However, these efforts are insufficient due to three reasons and the need for HCI concepts to evolve. First, resource constraints are increasing faster, which not only affects material and budgets but also the availability of experienced care and medical personnel. This situation implies that digital systems must take over labor and processes sooner than later and support the training of inexperienced staff, thereby increasing the complexity of HCI. Second, technology itself allows for substantial changes in HCI as systems become increasingly autonomous in their analysis and decisions, even in communicating and interacting with one another. Consequently, tasks for and interactions with humans also change as their roles shift increasingly toward supervision and exception handling. Third, an increasing conflict between the required sustainability of digital systems and the increasing speed of hardware and software development, especially in the health care sector, likewise demands new solutions for HCI, including ad hoc revisions and updates addressing possible crises. 

This paper contributes to the literature in two ways: first, (a) three use cases for digital applications with their individual technical and organizational requirements are described for broader comparison and learning; and second, (b) the generalization of insights from the three cases supplies the opportunity to recognize shared challenges to all digital applications in health care, centered in the HCI field for the specific requirements of medical personnel. 

The structure of this paper is as follows. Section 2 describes the conceptual framework from the management, medical, and computer science perspectives as a common background for the use cases. Section 3 outlines the specific analysis and development work implemented for the use case emergency medicine (University Medical Center Münster). Section 4 and Section 5 respectively describe the use cases on patient administration and cancer diagnosis at the University Medical Center Göttingen (UMG). Section 6 discusses the similarities and differences of the use cases and the generalized learnings regarding HCI. Section 7 concludes this paper and describes avenues for further research. 

## 2. Conceptual Framework

### 2.1. Human-Computer Interaction

**Figure 1:**
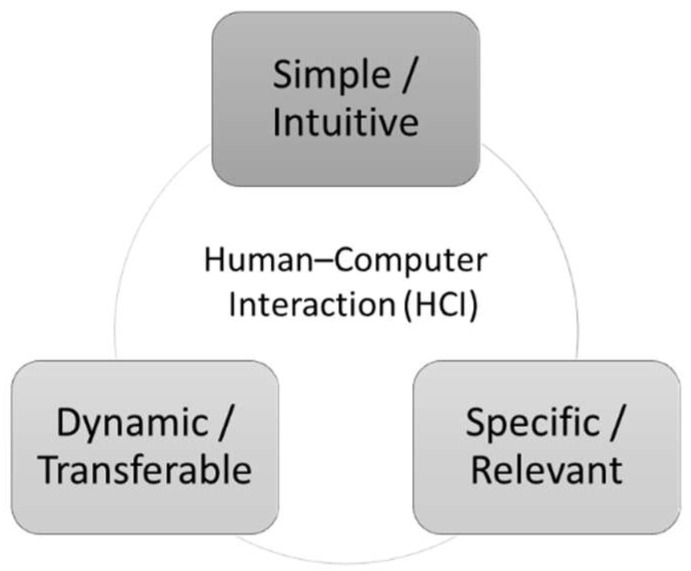
Standard HCI requirements.

HCI research is defined as “a discipline concerned with the design, evaluation, and implementation of interactive computing systems for human use and with the study of major phenomena surrounding them” [19]. It typically revolves around a set of four key issues that shape and are shaped by the interactions of individuals with computing systems [20]. The first important ingredient in HCI research is the technology itself. Here, input and output devices and the different interaction styles are discussed as material properties of the respective technologies [21,22]. The second key issue involves the tasks in question, as the jobs where technologies are used vary in complexity and goals [23]. The third concern is context, which can be differentiated according to regional, organizational, or social circumstances [20]. The fourth and final concern in HCI research is the human aspects, such as demographics, cognition, or motivations of individuals. The aforementioned key issues manifest in the particular interaction that is central to HCI studies. Here, research differentiates between what happens before technology introduction (i.e., design) and afterwards (i.e., impact). For example, Diederich et al. develop design principles [24] for a conversational agent as a new arising technology format to be used in enterprises. In this context, the technology, represented by the conversational agent, determines the design of an interaction that enables tasks in companies (e.g., a recruiting assessment) in a professional business context. Here, the HCI should be perceived as natural and human by the user as much as possible so that an intuitive communication flow is created, that is, a digital interaction with a human being. Impact studies also address the four key issues of HCI. For example, Goh et al. examine the effectiveness of advergames in online advertising campaigns [25]. They examine the use of this technology as a unique advertising strategy (task). Specifically, the HCI elements of interactivity, fit, and expectancy are evaluated as a function of human factors (e.g., attitude toward advergames, attitude toward brand, and purchase intention). Applying this generic HCI framework to contemporary contexts requires important considerations. Regarding technology, note that due to technical advancement, including increased storage and processing capacity, broadband internet accessibility, and availability of big data and advanced forms of analytics, the diversity of relevant devices has substantially increased as well. At the same time, the affordances of these technologies have extended, ranging from simple information storage to intelligent and evolving prediction sand visualizations. Accordingly, the tasks where computing systems play a role now cover a broad range of jobs involving administrative, service, and knowledge work. In a similar vein, through digitalization across societies, the variety of contexts in which technology use is situated have crossed the borders of organizational office spaces [26]. Finally, the omnipresence of easy-to-use and readily accessible technologies casts human factors in a different light. While computing systems in the past were only handled by technology specialists and administrative personnel, technology is now used by everyone and everywhere [27,28]. HCI research in the health care sector has only partially attended to this new reality. In particular, though progress has been made in the interactions involving doctors and intelligent systems in medical treatment processes [11,29,30], the steps preceding these processes have received very little attention. Closing this gap is important, because a key insight from existing research on health care IT adoption is that, besides data management issues and organizational hurdles, existing applications do not cater enough to the specific needs of the human stakeholders who are supposed to interact with the systems [31,32]. This finding leads to the general HCI model in Figure 1 below. 

### 2.2. HCI Regarding the Front End

Several features like the multitude of actor groups inside and outside the hospital and the interaction of a large number of IS make the HCI requirements in the front end of medicine (before patient treatment) special (Figure 1). As various persons and systems are included, HCI design should be very simple and intuitive. Furthermore, transferability is required in the dynamic environment of different systems and actor settings, for example, the transfer of patients from local or emergency doctors to medical centers. Finally, the HCI should foster the recognition of specific and relevant information given that the time and resources for data transfer are usually limited yet further medical procedures depend very strongly on crucial information sharing. This requirement leads to the request and practical implication to analyze the involved processes and specifications carefully and in great detail. The three following application cases for digital applications in this area highlight this general expectation to allow a generalized analysis of HCI concepts in the front end of medicine.

## 3. Use Case A: Digital Support for Emergency Medical Services (Münster)

### 3.1. Background

The interface between emergency medical services (EMS) and inpatient care in emergency departments (EDs) is of major importance to the front end of medicine before patients are treated [33,34]. It is characterized by a multitude of medical, structural and organizational aspects. Patient transitions from EMS care to the ED in hospitals are known as high-risk events for medical errors. Scientific analyses of medical treatment errors often show coordination and information problems between pre-clinic and clinic during patient handoffs [35]. Four key potential ways are identified to improve the structure and process of patient handoff [36]: (1) communicate directly with the hospital-based physician responsible for patient care; (2) increase interdisciplinary feedback, transparency, and a shared understanding of the scope of practice between intra and extra hospital persons; (3) standardize some handoff aspects; and (4) harness technology to close information exchange gaps. Several research results show the particular importance of technical solutions to improve structures and processes in patient transition. The major issue, for instance, the availability of clinic personnel at the time the patient arrives at the clinic to take over the patient, forms the EMS. Distinctive clinical duties and non-overlapping sites of work are leading to potential communication and teamwork gaps, which may be especially costly in the care of critically ill patients [37]. It is important that the entire team stays alert in the time required to take over the patient in a protected environment at the right time. A case study analysis of air rescue services that carried out 237 transports to a maximum care hospital revealed that 15.6% of all the cases were connected with time delays in the hospital. These delays resulted from waiting times for the physician to continue the treatment or for other team members in the resuscitation room [38]. Unfortunately, the availability of all team members is required directly when the patient arrives. 

### 3.2. Medical Requirements

Successful EMS depends on fast, high-quality care and the effective communication between all participants. As opposed to other countries, EMS in Germany includes the care of emergency doctors at the site of the emergency. Typically, paramedics are the first EMS to arrive at the emergency site, followed by an emergency doctor. Emergency doctors must have completed special training and have experiences in a relevant medical specialty, such as internal medicine, surgery, or anesthesiology. Once the emergency doctor has assessed and stabilized the patient, it is crucial that the patient is quickly transported to the nearest appropriate hospital. Clarke et al. show that after 90 min the mortality of patients with abdominal trauma increases by 1% every 3 min [39]. The selection of the nearest appropriate hospital is likewise crucial. A recent Canadian study by Fleet et al. shows that trauma patients treated in rural EDs have a higher mortality rate and are more likely to die pre-hospital or in the ED compared with patients treated at an urban trauma center [40]. Therefore, pre-hospital emergency care faces the challenges of providing high-quality medical care and solving time-pressing logistic issues. At the same time, the targeted hospital must be prepared to treat the patient as fast as possible. It needs information on the patient, the current state, relevant medication, and so forth. In addition, it needs to know the estimated time of arrival of the patient in order to have everything in place once the patient arrives. Trauma care requires different medical specialists, such as orthopedic surgeon, radiologist, anesthesiologist, and neurosurgeon. Since these specialists are busy, any waiting time for them must be avoided. Currently, the emergency doctor calls the hospital physician by phone to describe the state of the patient and share other relevant information. No automatic exchange of data transpires between EMS and the targeted hospital. After the phone call, the hospital knows the guessed time of arrival but not the estimated time based on the current location of the ambulance car. Any discrepancy between guessed and actual time of arrival means either more waiting time for the medical specialists (too late) or the danger that not all specialists will be available when the patient arrives (too early). Unfortunately, a transport delay cannot always be avoided (e.g., should the patient status deteriorate). As the emergency doctor is busy treating the patient in such cases, the hospital is not always called and informed about the delayed arrival.

### 3.3. Application Concept

**Figure 2:**
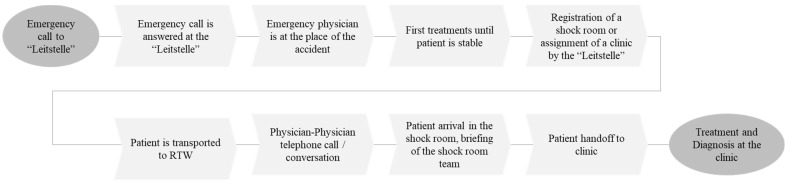
Standard emergency process.

A detailed process analysis is one of the most important tasks of a digitalization conception phase, especially in health care. On the one hand, the process analysis shows a complete and consistent understanding of the rescue and emergency process. On the other hand, the detailed analysis forms an important basis for the requirements analysis and provides important preliminary results for an application development. The following approach is used for the entire process recording: (i) determination of relevant processes/cases, (ii) process recordings in the form of expert workshops, and (iii) annotation of identified user requirements. To determine the relevant processes or cases, different statistics on severely injured persons are analyzed. Within the statistics, the number of serious injuries is examined according to certain injury patterns. A suitable case categorization proposal is the injury pattern of the TraumaRegister DGU. The annual report of TraumaRegister DGU determines the frequency of various injuries in nine body regions with a severity of at least 2 points (severe injuries are given 3 points on the Abbreviated Injury Scale, see descriptions at https://eena.org/advanced-mobile-location-aml-report-card (accessed on 17 September 2022).; http://www.traumaregister-dgu.de. (accessed on 17 September 2022)). This frequency defines a certain injury pattern, which represents the relative distribution of injury frequencies in certain body regions. To achieve high coverage during the conception phase, the following relevant cases could be identified using TraumaRegister DGU: head, thorax, extremities/spine, and fractures. Based on this, a process analysis method is used that conveys a comprehensive and complete process overview and creates a vitalization for all participants and the requirements analysis. In addition to the visualization of the whole rescue process, associated information flows are included. The recording of these steps follows the time axis with a chronological and functional time reference. Subsequently, the recorded process is represented and used as basis for annotations for improvement (Figure 2). 

### 3.4. Functional and Technical Requirements

To transform stated workshop results into requirements for a potential software system, the results were analyzed, evaluated, and converted into user stories. User stories are short representations that describe the requirements’ role, goal, and potential benefit on a higher level. These user stories were later partitioned into detailed tasks within the software development process [41,42]. Within this digital development, derived user stories were prioritized and evaluated with regard to the effort required for the technical implementation. From this prioritization, user stories could be classified in different categories representing core functionalities of the potential software system: (1) the collection and transfer of emergency information, (2) the transport decision, (3) the visualization of information, and (4) non-functional requirements. Thus, the following user stories demonstrate the core requirements of the potential software system. 

During the workshop sessions, participants frequently stated the need for optimized communication between those involved in the emergency chain. Here, the exchange of information between the emergency doctor on site and the specialist in the clinic was described as particularly relevant. Specifically, the structured recording and transmission of emergency data was emphasized, which should improve the coordination between involved parties and prevent erroneous information exchange. These results are represented in the following user stories:

- “As an emergency doctor, I would like the information about the patient and the emergency to be transmitted directly to the hospital without any loss of information in order to shorten the doctor–doctor consultation and avoid discussions.”
- “As the resuscitation room leader, I want the information to be exchanged between the emergency doctor and myself in a structured digital way so that as little irrelevant information as possible is exchanged and no errors occur.”

A digital system should therefore enable a structured and loss-free information exchange between the emergency doctor on site and the target clinic. The potential system should allow emergency information to be recorded and then transferred to a target clinic where the data can be accessed. Besides information exchange, emergency doctors formulated the need for support in crucial decisions. In detail, the transport decision was described as particularly challenging because a variety of information has to be considered, information that is usually not explicitly available but based on subjective assessment.

- “As an emergency doctor, I would like to be able to see the capacities of the surrounding clinics with a relevant competence profile in order to be able to decide which clinic is suitable for the transport decision.”
- “As an emergency doctor, I would like to be supported in the selection of the clinic by information such as the travel time to the clinic and the treatment focus of the clinic so that this decision is not made purely subjectively.”

A digital system should display relevant data to objectify the transport decision. These data should primarily include the clinic capacities, treatment focus, and distance based on the user’s position. As another core functionality of a digital system, it was formulated that the collected data should also be made accessible to the entire resuscitation room team to prevent extensive handover briefings and search for information. A technical solution should provide emergency information to the whole treatment team (e.g., by visualizing this information on a central display). On a technical level, the system must integrate a functionality to transfer information to a display within the resuscitation room. In case of multiple resuscitation rooms within a clinic, the possibility of selecting a resuscitation room from a list should be integrated.

### 3.5. Concept Outline

**Figure 3:**
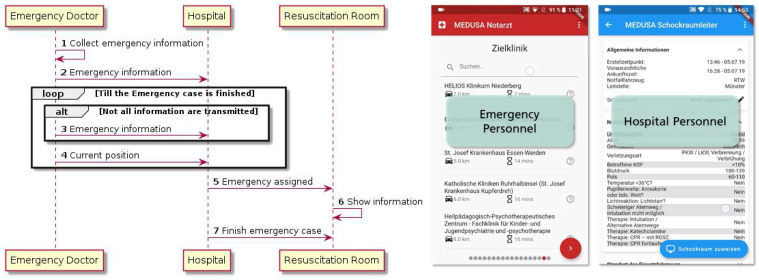
Simplified emergency process using digital applications.

To come up with a solution for the requirements specified during the workshops, three distinct applications were created: (1) a smart phone application for the emergency doctor, (2) a smart phone application for the hospital staff (specifically, the resuscitation room leader), and (3) a web application to be deployed in the resuscitation room itself. Figure 3 shows a simplified version of the emergency process using digital applications. Step 1 consists of the collection of emergency information in the application by the emergency doctor. We decided to implement this by asking a set of questions developed by the medical partners and providing answer categories for each question. 

One example would be the question about the patient’s current heart rate, which could be answered with one of three options: <60, 60–100, or >100. We decided to follow this idea instead of giving the emergency doctors the option to enter free text for two reasons. First, it helps focus on valuable information instead of unnecessary details (e.g., whether the current pulse is 55 or 57 is not important). Second, it helps speed up the information-gathering process. After the emergency doctor decides which hospital he wants to approach, the collected information will be transmitted to the hospital (Step 2). This decision is supported by the application by showing the certification level of the hospital issued by “TraumaNetzwerk DGU” and the capacity of the hospital. 

Starting from the beginning of the information transmission to a certain hospital until the emergency is manually finished—normally when the emergency doctor hands over the patient to the hospital staff—the application tries to send yet-unsent information continuously (Step 3) and submits the current location of the emergency doctor to the target hospital (Step 4). The resuscitation room leader will be notified of the incoming emergency through push notifications in the mobile phone and can view the submitted information. The emergency case can also be assigned to a monitor mounted in the resuscitation room where the information is displayed for the whole team (Steps 5 and 6). The information is presented until the team leader marks the emergency case as finished (Step 7). 

### 3.6. HCI Reflections

HCI concept developments were included in the digital application, especially in the speedy inclusion of EMS data at accident sites for emergency personnel before patient transportation to hospitals. Speedy and exact data acquisition from personnel into a digital app for delivery to the addressed hospital were of central importance in providing the least hindrances to EMS personnel and, at the same time, maximum information and preparedness options for the receiving hospital staff. 

## 4. Use Case B: Inpatient Information Management with Automated Information Extraction (Göttingen)

### 4.1. Background

While a centralized platform (e.g., a nationwide electronic health care record, EHR) for patient data is envisioned to enable data exchange between different health care institutions and platforms [43], analog data exchange is the norm in everyday work. Poor interoperability in health care [32] results in process efficiency deteriorations. Medical staff, for example, are required to scan documents such as external reports and doctors’ letters and manually enter the essential information into the hospital IS. This manual process consumes valuable working time and leads to a high rate of transmission errors [44]. The treatment of malignant tumors depends on the individual characteristics of patients and tumors. In many cases, imaging data, laboratory findings, and molecular-biological tumor characteristics are considered. Furthermore, the patient’s general condition, as well as his or her personal goals and wishes, must be considered. Most tumors are not treated with just one modality (for example surgery), but multimodally, which requires a multidisciplinary discussion of each patient’s case before any treatment. Relevant data from physician’s letters, pathology findings, laboratory findings, operating theatre reports, and functional examinations from the currently treating and previously treating health care provider need to be collected, structured, and presented at multidisciplinary tumor boards. Information transfer from primary care to specialized tumor centers is currently performed with unstructured electronic or paper documents–even though a relevant fraction of data itself is delivered in a structure itself (such as laboratory findings). These documents are usually not read out automatically. Therefore, identical data from unstructured findings must be entered several times into hospital information systems. In the case of a tumor patient, this can be for the admission findings, the registration for a multidisciplinary tumor board (TB), the registration for a consultative examination, the doctor’s letter, but also specialized registers for clinical research or in databases for translational research. 

Last but not least, diagnosis and treatment data must be coded for billing in the DRG system. These repetitive entries are time-consuming and error-prone. This case study aims for automated data extraction of routine findings from non-structured PDF documents. As a proof of principle, the extraction of the TNM tumor formula from a pathology finding was to be carried out. The TNM classification describes the size of a tumor (T), as well as the involvement of lymph nodes (N) and the presence of metastases (M). Furthermore, characteristics of the local growth behavior are coded (e.g., infiltration into vessels(L), invasion into vein (V), completeness of operation (R)) [45]. Colorectal carcinomas are among the most common malignant tumors in Europe and the USA. The TNM classification has a significant influence on multimodal treatment. Therefore, the extraction of the TNM classification for colorectal carcinomas was obvious. The aim was to show whether automated data extraction is subject to frequent errors and to what extent, if any, higher efficiency is shown here. As an alternative to the procedure mentioned here, it could be pointed out that the use of structured reports should be implemented. In principle, this is correct and points to a deficit of current IT systems. Nevertheless, the use of structured documents is not current practice—even the German national health record initiative (“Elektronische Gesundheitskarte”) does only prescribe certain document formats but not the use of specific structural elements within the stored documents (e.g., use of TNM in pathological reports). Furthermore, a structured report can only capture known factors, whereas an automated data extraction gives the additional possibility to extract further data from unstructured reports and correlate them with the known risk factors.

### 4.2. Functional and Technical Requirements

To identify the relevant functional requirements of a potential digital solution to the described problem, focus group discussions and interviews were conducted with experts (medical specialists and IT specialists) from the UMG. Furthermore, literature was identified that underlines the aspects of the experts and provides approaches for technical realizations. Overall, three requirements were identified. First, process automation through digital software eases the workload of medical personnel who would otherwise have to complete these processes manually. The manual transfer of data into IS, even though these data already exist, is an example of such a process. Media disruption causes medical staff to transfer the information they need into multiple digital systems until now. Experts indicated that manual data entry takes up to 5 min per patient, time which could be used to interact with the patient and improve the quality of care [46]. Therefore, a digital solution should overcome this media disconnect and be able to identify and automatically extract specific text fragments from an image file (e.g., scan) and integrate them into an existing software solution. This would eliminate the need for medical staff resources for such a work process and thus increase the time efficiency of the medical staff. Second, the information in text format accounts for an estimated 40% of patient data stored in clinical IS [47]. Medical texts contain important information about prescriptions, symptoms, and findings relevant to treatment decisions [48]. However, the manual transmission of text fragments carries the risk of transmission errors. Especially in the case of text codes, this can have fatal consequences, as the erroneous transmission of even a single letter can affect the patient’s diagnosis and treatment. Human error in the transfer of data is approximately 13%. Text mining approaches provide an opportunity for more accurate results and fewer transcription errors [49]. Such algorithms automatically extract concepts, objects, and events from the text as well as their relationships and associated attributes [50,51,52]. In health care, statistical learning approaches such as support vector machines and logistic regressions have been proven to extract information from continuous text [53]. 

For information occurring in fixed patterns, such as TNM codes, pattern recognition and regular expressions (regex) have proven to be very efficient [54]. In Germany, the General Data Protection Regulation stipulates that automated decisions must be traceable for certain areas of application. The processing of patient data and medical diagnoses are an explicit part of this (Art. 9 (2) (h), Art. 9 (3) DSGVO). This stipulation has limited the use of AI in the health care sector and led to uncertainty among users. Therefore, the third requirement is to design the automated applications and use of AI in such a way that users can explain the results to themselves [55]. Users are more likely to use systems with decision-making processes they can understand [56]. Particularly important is the reason for a decision and the possibility of influencing the results [57]. The property of understanding the internal processes of an algorithm (e.g., AI) and describing it in a way that is understandable to a human is called explainability [58]. Cutillo et al. suggest visualizing attention markers and certainty levels of results for explainability. For text-processing algorithms, this can be achieved by marking the outcomes in the corresponding input texts [59]. Accordingly, users are shown which text modules have been incorporated into the result and how. The explainability of AI depends on the algorithm chosen. While rule-based approaches already feature explainability in the rules, more complex systems like neural networks are so-called black-box models, where no statement can be made about how the model made its decision. A conflict between transparency and performance of an algorithm becomes apparent [60]. One way to use more powerful models and ensure explainability is a human-in-the-loop approach [61]. In this approach, the results are presented to and monitored by an expert. The resulting new data and insights can be used to optimize the applied algorithms.

### 4.3. Concept Outline

**Figure 4:**
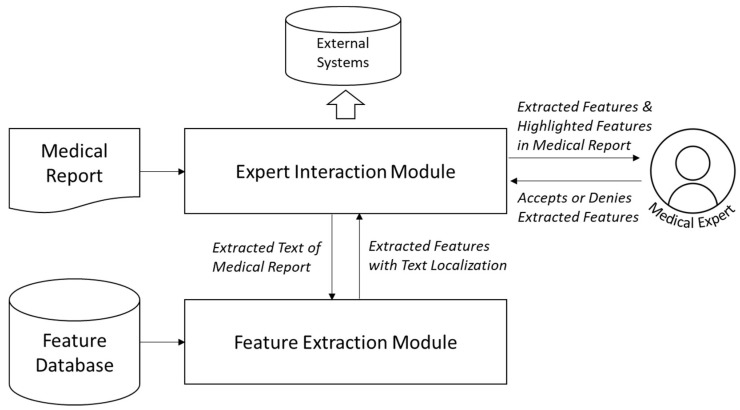
Simplified interaction of modules for an automated extraction tool.

A concept was developed consisting of an expert interaction module (EIM) and a feature extraction module (FEM) (Figure 4). The EIM digitizes the medical reports, communicates the extracted text with the FEM, and visualizes the results of the extracted features through an interactive user interface. The FEM analyzes the text by identifying the relevant features captured in a database. If the medical experts correct the results of the extraction, this information will be considered for future analyses so that a supervised learning approach leads to the continuous improvement of accuracy. The extracted features can be further processed by passing them on to external systems. In close collaboration with the medical experts, the following parameters were extracted from the pathology findings: T, N, M, L, V, R, the number of lymph nodes removed (X1), the number of lymph nodes affected by the tumor (X2), and the features neoadjuvant therapy and tumor localization. 

### 4.4. Expert Interaction Module

**Figure 5:**
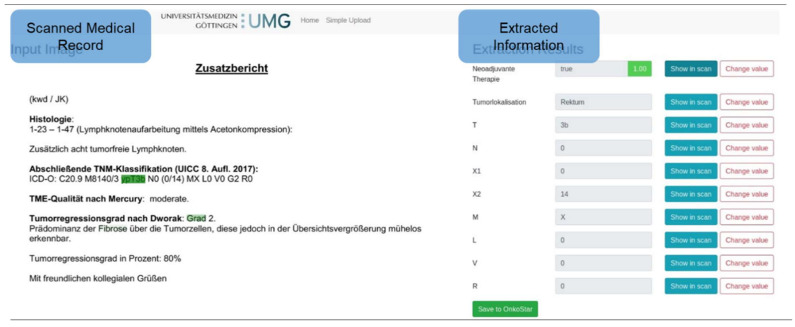
Screenshot of the user interface (left: scanned pathological finding; right: extraction of TNM and ICD codes).

The EIM implements the complete pipeline from analog paper-based reports to visualization of the automated extracted features that can be validated by the medical expert. In the first step, the paper-based medical report was digitized through a scan. This step is a common standard because the documents are usually saved as pdf attachments. Second, the digital document was converted into a text document through optical character recognition. In this text format, the data were forwarded to the FEM for information extraction. For the visualization, the EIM expects the individual features with their extracted values from the FEM. Each feature has, next to its value, a list of information relevant to the prediction (e.g., the original pixel values in the scan and, if applicable, probabilities for the certainty of the prediction). A user interface (Figure 5) was designed to visualize the results. On the right side of the figure, the extracted information is displayed, and on the left side, the original document is shown along with the respective points of origin of the extraction highlighted. The user can select individual values and specifically trace the localization of individual extractions. If no clear extraction is possible for individual features or the prediction is too uncertain, then these values will be highlighted and the user will be prompted to validate or complete the values. This process allows a consistent tracking of the automatic decisions and a simple manual validation. In case of a wrong extraction, the user can correct the result. This information is forwarded to the FEM for future extraction analyses. The final extracted features are exported and can be sent to databases or other IS so that a connection to external systems is possible. 

### 4.5. Feature Extraction Module

The FEM extracts the features from the text identified from the pathological finding through the EIM. In previous studies, regular expressions and non-rule-based algorithms, such as support vector machines, logistic regressions, and decision trees, were identified as robust methods for extraction from medical texts [53,54]. In the investigated case, feature extraction required a rule-based pattern matching approach. In this process, the individual parameters of the TNM and ICD classification runs were optimized and the algorithm could be applied to a test set of pathological findings. For the feature tumor location, T, N, M, X1, X2, L, V, and R, a model with corresponding regex for the individual parameters was developed. To achieve high sensitivity for keeping the number of false detections as low as possible, the regex for the individual parameters was derived directly from the range of values of the TNM classification [45]. For the tumor localization values, the ICD code was first extracted and then assigned to the corresponding technical terms; in the case of colorectal carcinomas, only the tumors of the digestive tract (range: C17.0–C22.1) were of interest. The text of the documents was analyzed according to the regex. In case of a hit, the recognized value for the characteristics was stored. In case two different values were detected, the value for the feature was set to “ambiguous value” to request validation by the expert. Thus, the FEM returns three types of values to the EIM: (1) concrete value (class 1): the pattern resulted in exactly one hit and thus a unique value can be returned, (2) ambiguous value (class 2): the pattern resulted in more than one hit and thus no unique value can be returned, and (3) empty string (class 3): the pattern resulted in no hits and thus no value can be extracted, or an X is extracted (for M, L, V, and R the pathological findings contain as “X” if no medical statement can be made). For the extraction of the feature “neoadjuvant therapy,” a machine learning (ML) approach with logistic regression was used. The logistic regression received as input values a frequency matrix of the texts to be classified (bag of words, BOW). The logistic regression was trained with the BOW of the texts. To make the result as unambiguous as possible and for model generalization, the L1 regularization (lasso regression) was applied. Based on the ML approach, the FEM returned the class probability visualized in accordance with the certainty (green for *p* ≥ 95%, yellow for *p* ≥ 90%, and red for *p* < 90%) (Figure 5). The feature extraction was tested with unstructured pathology findings of patients (62.7% male; 37.3% female) with colorectal carcinoma. Inclusion criteria were as follows: patient age of 18 years or older (21–96 years), colorectal carcinoma in terms of a primary surgical case of the colorectal cancer center, corresponding data in the tumor documentation program Onkostar, histopathological findings of tumor resection from the UMG, and patient’s consent to data analysis for scientific purposes in the context of the treatment contract at the UMG. Based on these inclusion criteria, a total of 247 patients with colorectal carcinoma who were treated at UMG in 2017 and 2018 were included in the study, resulting in 430 pathological findings for extraction analysis. The study was approved by UMG ethics committee and data protection officer.

### 4.6. Results

To obtain a comparable result for the automated feature extraction, the same pathological findings were manually coded by a medical postgraduate student. The student coded the data twice (this data set is referred to as “Manual Coding”) to ensure validity. This coding was expected to represent the comparable truths. However, the comparison of both data sets, indicated that even the manual coding included a few errors (Table 1). The correct information was evaluated through an overlay of the data sets. The feature “tumor localization” was removed from the evaluation as the mapping between ICD codes and localizations caused discrepancies of definitions and medical assessment.

Overall, the results show that the automated transmission results in an accuracy of 93.5% for complete data, however the individual features are extracted with an accuracy of 97.5% or higher. For further evaluation, the absolute errors on an aggregated patient level were compared. Table 1 lists the accuracy of the correct identification of a feature (class 1) while Table 2 includes the cases in which the algorithm indicated an ambiguous value (class 2) for manual expert validation. 

The EIM expected a validation of the extracted feature in 9 cases, where the algorithm examined ambiguous results. Therefore, medical personnel were only required to manually assess the finding of 9 patients. In comparison to the current procedure, where medical personnel manually copy the information for all cases, the automated transmission is a tame-saving and accurate alternative. The results provide a proof of concept for the automation of feature extraction based on text mining methods (regular expression and ML algorithms) as an alternative to error-prone, repetitive manual evaluations. However, the evaluation of the algorithm was conducted through a small sample of pathological findings and should be validated through a larger sample. An extension of the data pool could improve the results here on the one hand and enable a broad application on the other hand. An evaluation of additional documents, for example for other tumor entities, could lead to a further improvement of the algorithm as well. This use case shows that an automated data extraction with sufficient reliability is possible with reasonable effort and could be useful for clinical routine and research. Further development of corresponding algorithms with transferability to findings from different practice management systems and hospital IS could contribute to reducing the documentation effort and improving the data usability for clinic and research.

### 4.7. HCI Reflection

The described information from medical reports is of high importance for medical staff and must be equally and accurately available in each of the used systems. Hence, it was crucial not to develop a black-box solution for automation but to integrate an interface for simple verification and validation. The user interface is intuitive to use and dynamically displays the relevant points in the existing findings. The supervised learning approach based on user feedback ensures that the physicians improve the algorithm and thus learn to trust it, with the result that, at a certain point, manual validation and interaction are only necessary in a limited number of cases.

## 5. Use Case C: Cancer Diagnosis (Göttingen)

### 5.1. Background

The complex and challenging case of head and neck tumor treatment often affects bone, soft tissue, and the tumor microenvironment at the invasive edge of tumor transition [62]. Head and neck squamous cell carcinomas (HNSCCs) show an incidence of about 600,000 cases worldwide per year. Unfortunately, the mortality rate is equally high at 40–50%. Although the tumors originate in the relatively small area of the upper airway, oral cavity, pharynx, and larynx, it is a highly heterogeneous malignant disease. This is due in part to the complexity in embryological development demonstrating a large variety of molecular changes. As the knowledge on the molecular mechanisms of HNSCCs progresses, the understanding of this disease and its characteristics is currently undergoing continuous changes. Therefore, a multidisciplinary approach is often required to apply possible therapeutic options and assure successful treatment. While smoking and excessive alcohol use remain the classical risk factors for HNSCC, currently, infections with high-risk human papillomaviruses (HPVs) have been shown to cause a substantial and rising proportion of these tumors. Often, depending on whether the patient is HPV negative or HPV positive and their socioeconomic, clinical, and molecular profiles, the prognoses of the tumors differ and become more complex to predict. Such complex tumors may come with an increased risk of surgical complications, toxicity, and other adverse outcomes during treatment, including prolonged hospitalization, functional and cognitive decline, and death [30]. To further optimize clinical outcomes, it is important to identify individual molecular drivers and treatment requirements for interdisciplinary health care teams and caregivers to assist with decision making and guide supportive interventions during treatment. However, the number of cancer driver genes in HNSCCs is exploding, and their influence on the epithelial-mesenchymal transition of the cancer microenvironment requires careful interpretation. The disciplines involved in current tumor treatment have to include these findings in their treatment plans. Often diagnosed by dental professionals, the complex anatomical area requires patients who suffer from a malignant tumor in the head and neck region to be treated in specialized medical centers. Here, cranio-maxillofacial surgeons together with otorhino surgeons plan and execute surgical removal and plastic reconstruction of malignant tumors in the head and neck region. Guideline-driven treatment plans usually follow a primary biopsy of the tumor to determine the pathologic tumor type. Early in the process, these findings are evaluated in a TB set up to determine surgical and oncologic interventions depending on the clinical staging of the tumor. Here the surgeon’s clinical evaluation is combined with medical imaging and an initial pathological evaluation. The successful management of head and neck cancer now requires a cooperative strategy among a broad group of medical disciplines, including head and neck surgery, radiation and medical oncology, imaging, clinical pathology, molecular biology, social and psychological work, and nutrition specialists. Translation of continued advances in these fields by cooperative work will continue to yield incremental advances in diagnosis, staging, treatment, follow-up, supportive care, and quality of life [63]. Today, TBs are universally adopted, and the increasing complexity and specialization on the delivery of precision medicine for head and neck cancer require updated and diverse opinions [64]. Here the TNM classification plays a pivotal role in clinical care, clinical trial eligibility and stratification, research, and cancer control. Today, the staging of head and neck cancer has evolved in response to an enhanced understanding of tumor biology, disease behavior, emergence of new diseases, and better prognosis as a consequence of improved diagnosis and management. Following a TB’s recommendation, treatment plans are executed, thoroughly reevaluated, continued, or changed. However, the growing complexity of HNSCC tumor treatment has required further adaptation of the TNM introducing new classifications in recent years and increasing the complexity of tumor staging. Evolving with the change and progress in targeted therapies, cancer diagnostics, and surgical advancements, treatment groups must constantly catch up with, evaluate, and review the latest clinical trials and information on cancer diagnosis and treatment as well as adapt to potential advantages while coping with the limitations associated with them.

### 5.2. Functional and Technical Requirements

Such multilateral and complex approaches lead to interlocking medical treatments that are effective particularly for head and neck cancers [63,64]. However, necessary specialists and resources may not be readily available or inadequate in remote or local hospital situations [64]. Consequently, local treatment options become limited and larger treatment centers may be overburdened by transferred patients pushing the productivity limit of health care systems, as was observed during the COVID-19 pandemic [65]. Attempts have been made to overcome the disadvantage of a single-institution TB through central conferences involving multiple institutions. However, the accessibility to such conferences is limited due often to their locations and the need to factor in travel time, which nowadays might even be completely impossible because of pandemic-related physical distancing. While web-based TBs may complement local treatment teams, their accessibility is rare due to the challenges of secure web-based medical conference platforms [64]. Participation patterns may also vary between the involved disciplines. To overcome this challenge and improve the outcome of TB, patient survival, and process measures [66], current applications focus on a human-centered approach combined with ML models to help improve treatment quality and process efficiency for physicians simultaneously [67]. The impact of health information systems (H-IS) holds the potential of deciphering the complex distribution of molecular, clinical, and patient-specific tumor drivers in cancer data analytics. Recent innovations in molecular analysis, 3D reconstruction, and oncologic therapies have increased the complexity of conjoint therapies. Therefore, it is imperative to identify significant data while at the same time focusing on the volume and breadth of individual and collective data [67]. To improve cancer treatment, attempts have been exerted to harness the power of EHRs and the potential of ML systems by using routinely collected data [68,69]. H-IS can help interpret the increasing amount of data from various molecular changes, specialized research, TNM classification, and an increasingly digitalized clinical process while avoiding unnecessary testing, excessive administration, and excessive cost for implementing innovative approaches [69]. Despite the positive acceptance and growing interest concerning big data, the evaluation of ML systems has shown that issues of data safety and security concerning patient identification, genetic information, informed consent, and data sharing remain critical and challenging [70]. While the interest in ML has led to the impression that big data approaches can solve most problems, higher accuracy is needed to improve clinical impact because an evaluation of big unbalanced data can put clinicians and investigators on a costly, difficult-to-secure, and unsuccessful path [67,71]. To improve the accuracy and, in turn, the clinical impact on oncologic patient care, ML analytic systems have to represent the oncologic system and identify significant data and correlations while promoting user training by guided data acquisition. The goal should be to support medical decision makers in complex treatment approaches, thereby yielding better results with improved treatment efficiency. ML models have often been suggested and applied to various problems relating to patient treatments. However, while the majority of IS health care concepts have taken advantage of EHRs, future H-IS models have yet to start touching the complexity of HNSCC with regard to TNM or even molecular classification. Such an evidence-based and contextualized clinical decision support system (DSS) has to be able to fulfill the following requirements:

1. find the most important attributes for treatment decisions,
2. predict an optimal treatment for a patient based on all available attributes, and
3. estimate the prediction accuracy.

### 5.3. Concept Outline

To address these requirements and include the increasing tumor complexity, a Division-of-Labor (DoL) structure can support DSSs and offer a framework for developing durable ML systems [72]. Such DoL frameworks have to reflect real-life conditions by using the TB system as a blueprint for a DSS. The frameworks comprise an “Expert” and a “Central Executive” (CE) that can treat complex analysis tasks as a composition of less complex sub-tasks. Such architecture resembles established decision-making processes at the TB level, where an interdisciplinary team gathers to combine subspecialties and make individual treatment decisions. To achieve a personalized treatment approach and avoid “cookbook medicine,” DSS-driven H-IS holds the potential to offer solutions that simultaneously help improve treatment quality and process efficiency for physicians. However, though a higher amount and variety of data may lead to more informed decisions, collecting these data requires massive investments in human labor, technical infrastructures, or organizational hurdles and capability gaps [73,74]. To resolve this inherent contradiction, the DSS of H-IS has to focus the analysis to uncover the most meaningful attributes and, building upon this foundation, restrict the data needed for the necessary decision support. Using data that are necessary to achieve satisfactory predictions, focused ML techniques, and balanced DSS can offer important support to the tumor treatment of HNSCC while avoiding additional administrative efforts or the need for technical infrastructures, which, due to the heterogeneity of health care providers, cannot be taken for granted in the majority of contemporary health care organizations. Accordingly, in cooperation with physicians who guided the research process and evaluated the outcomes, we designed an evidence-based and contextualized clinical DSS [75] for the treatment of patients with head and neck cancer by using an innovative ML framework, namely, the DoL framework [72]. This DSS keeps administrative effort at a low level by being able to function with minimal amounts of input data (i.e., medical staff is not forced to collect a set of mandatory patient attributes in order to receive a treatment suggestion), but at the same time uses all given inputs to increase its predictive power. The results indicate that our artifact could identify a reduced, case-specific number of meaningful attributes that retain sufficient predictive power. Furthermore, our artifact’s predictive component, comprising a standard classification model embedded in the DoL framework, outperformed standalone classification models. The treatment of oncologic patients is carried out in close cooperation with the University Cancer Center (Göttingen Comprehensive Cancer Center, G-CCC) as an interdisciplinary center of all the clinics and institutes of the UMG. The head and neck TB coordinate the interdisciplinary individual treatment plan in cooperation with the G-CCC to ensure the application of the latest research, diagnostic, and treatment protocols in cancer medicine as well as the close cooperation between hospitals and institutes in the G-CCC. To provide patients with optimal counseling and excellent treatment options, the G-CCC cooperates with other universities and its own research institutes in the national network of cancer centers. The close association with the German–Austrian–Swiss head and neck cancer group (DÖSAK) constitutes such cooperation. The DÖSAK database contains more than 4400 international oncologic records, of which 799 were contributed by the OMF Surgery Department of the UMG. The current registration is carried out according to § 65c BGBI. I S. Maintenance and assessment of the data are performed by technicians under the supervision of physicians involved in the oncologic treatment plan. All data evaluated in this study were fully anonymized prior to analysis. In the following, we refer to the collection of attributes belonging to an anonymized patient as “data elements.” Each data element is described by 248 numerical and categorical attributes—spread over 21 tables—concerning their demographics, illnesses, tumor diagnostics, and therapies. Besides data on treatment decisions, we consider only those attributes that are regularly collected before the treatment decision is made. The given data set comprises 38 such attributes related to the patients’ demography, medical history, and preliminary diagnoses. Therapies are represented by 6 attributes, indicating the therapy intention (palliative or curative care) and whether an operation, radiation therapy, local chemotherapy, systemic chemotherapy, or other therapy was carried out or declined by the patient. 

We develop a DSS that (1) finds the most important attributes for treatment decisions, (2) predicts an optimal treatment for a patient based on all available attributes (not restricted to the most important ones), and (3) estimates the prediction accuracy. In the following, we describe the components of the developed DSS.

**Figure 6:**
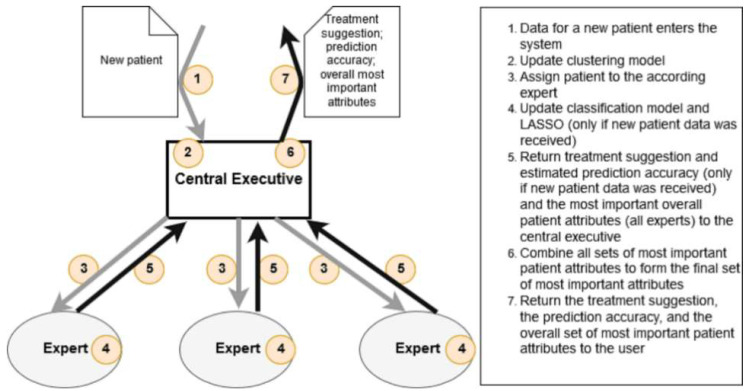
Division-of-Labor framework.

- Expert: We divide an Expert’s tasks into learning tasks, performed when the Expert receives new labeled data, and analysis tasks, performed when the Expert is queried for information. The learning tasks of an Expert are defined as (1) learning attribute importance ratings for each of the 6 treatment decisions and (2) modeling the relationship between the 160 features and the treatment decisions. Each time an Expert is provided with a data element, it updates its learning models for both tasks in an online manner and returns the suggested treatment decisions.
- Central Executive: Again, we divide the CE’s tasks into learning tasks and analysis tasks. The CE has a single learning task of performing patient segmentation and creating/updating an Expert for each segment. The analysis tasks involve (1) querying the most suitable Expert for a prediction and (2) identifying the most important attributes for decision making.
- Base Models: For our DoL system, we use of three kinds of ML models. First, we employ a feature selection method, namely, the least absolute shrinkage and selection operator (LASSO, [76]), to identify the most significant patient attributes. LASSO is a common method for feature selection that uses regularized regression to identify notable features (i.e., perform dimensionality reduction). Second, we use a binary classification model for the prediction of treatments. The last version of the DoL system employs a single type of classification. However, as part of our research, we try several classification models, both in a standalone manner and as a part of a DoL system, to find a model of high quality with respect to our measure of accuracy (i.e., the F_1 score). Third, for the purpose of task division, which is realized through patient segmentation, we make use of a clustering algorithm. Figure 6 illustrates our approach.

### 5.4. Results

As to requirement (1), the following Table 3 depicts the most relevant patient attributes for predictions. The scores of the most important attributes overall are their average over all prediction tasks, weighted by the number of samples in the respective test sets.

The attributes identified by the DSS are evaluated with respect to their validity by multiple physicians from the UMG. On the one hand, many identified attributes confirm existing theory on head and neck cancer treatments and are already used by physicians to make decisions, such as tumor-(T)-classification, lymph node-(N)-classification, and metastasis-(M)-classification. The TNM classification of malignant tumors, comprising a patient’s T-, N-, and M-status, was developed by the Union for International Cancer Control and is globally recognized as a standard for classifying the extent of spread of cancer. Derived from the TNM classification, head and neck tumors are grouped into clinical stages, an attribute that our DSS also identifies as a significant contributor to the treatment plan. On the other hand, the DSS identifies attributes that researchers and practitioners do not typically consider for making treatment plans, such as a patient’s reasons for the initial visit to the doctor, an attribute that has limited causal implications but is of statistical importance for treatment decisions, as well as attributes that have not yet been introduced into medical guidelines but have been the focus of ongoing research, such as the inflammatory response of tissues surrounding a tumor. The invasive potential of cancer is dependent on the tumor environment and its interactions at the invasive edge of cancer [62]. As to requirements (2) and (3), we compare the performance (i.e., the $F_{1}$ score) of our approach to the performance of some baseline models and other popular classification methods with and without the use of the DoL framework. We tune hyper-parameters for the LASSOs, logistic regression models, decision trees, and SVMs, both as standalone models and as base models in a DoL system, and for the DNN, only as a standalone model. For this purpose, we use an exhaustive grid search over reasonable ranges with threefold cross-validation to tune parameters such as regularization constants, kernels, splitting criteria, class weights, hidden layer units, and activation functions. We assess the expected generalization performance by using 10-fold cross-validation with the training set and test set containing 90% and 10% of the data, respectively, in each iteration (Table 4).

Generally speaking, the DoL system based on logistic regression achieves the highest performance. Note that this particular model was implemented in an online manner, as initial analyses revealed its superior performance in contrast to other models implemented as batch models. Noteworthy classification tasks are the recommendation of an “operation” and the recommendation of an “other therapy.” The former is the only task for which the prior classifier is not (significantly) outperformed by the other models. For the latter task, multiple classifiers are tied in terms of highest accuracy. This outcome can be explained by the fact that these attributes have highly imbalanced values; about 92% of the recorded patients have received an “operation” while about 98% have not received “other therapy.” Classifiers, therefore, tend to recommend an operation and no other therapy for every data element. The DNN generally performed poorly. This poor performance can be explained by the small sample size, which is generally not suitable for highly complex models such as DNNs. We developed an intelligent DSS that is evidence based and makes situationally relevant recommendations [75]. It achieved the goal of supporting decision-making processes by providing treatment suggestions while avoiding the need for additional administrative efforts for medical staff by designing the DSS in a way that allows utilizing all patient attributes provided by the user while being able to function with the smallest sets of inputs. Specifically, the DSS does not require any input at all in order to function, but its ability to make informed suggestions increases with the number of provided attributes. In combination with the prediction accuracy provided by the DSS, physicians are given sufficient information to decide whether to accept or deny a suggestion. Furthermore, the DSS provides a list of attributes significant to the decision-making process—serving as a hint rather than an obligation—that can be used by medical staff to focus the data collection process. Therefore, administrative, technical, and organizational hurdles can be reduced while analytical and predictive power is retained. At the same time, physicians can derive new insights on the importance of specific factors, leading to increased learning based on evidence [77] and potential improvements of care through their integration in clinical workflows [78]. As a result of this approach, our artifact yielded results that are reasonable and insightful from the viewpoint of clinical practice in oral and maxillofacial surgery. In cancer treatment planning, significant patient attributes must be identified and correlated while unnecessary information and inefficiencies are reduced to allow cancer centers to offer fast and successful patient-guided treatment plans. Our DSS identified a set of attributes significant to treatment recommendations. Out of these attributes, most are aligned with existing guidelines while some have not yet been introduced into medical guidelines but have been the focus of ongoing research, such as the inflammatory response of tissues surrounding a tumor. Following the initial success of immune- and inflammatory-modulating therapies, inflammation and immunity are expected to become important targets in patients with cancer in the future. Moreover, to utilize data analytics in health care, it is essential to overcome the challenges of heterogeneous, inaccurate, and incomplete data structures, real-time environments, and the resistance of health care workers to changing technologies [79]. Using the DoL framework, we simulated the underlying analytic framework representing the collaborative characteristics of the medical experts involved in the treatment planning session of head and neck TBs, thereby reaching a higher degree of contextualization of the artifact [46].

### 5.5. HCI Reflection

The surgeons within this case clarified that they do not want to have to use another IT system as they were very cautious about potential distractions from their work and further administrative efforts. Nevertheless, they were very interested in the outcomes the system provided. These outcomes were primarily numerical and categorical (see tables above). The surgeons perceived the insights generated by the system as starting points for further discussions or even new research projects. Therefore, no graphical use interface has been developed. Instead, the results of the system were delivered to surgeons upon request in the form of tables and graphs with no further interaction possibility. Accordingly, what emerged was a more indirect and cognitive interaction, not a direct and technical interaction between the system and the human experts. The surgeons found that the system’s value does not lie in the automation of the decision processes around treatment recommendations. They had the feeling that, in the best case, the system could provide what they already do. Instead, the human experts were fascinated by unconventional findings of the system that were surprising. These outcomes of the system were then discussed in the group of surgeons and could trigger further investigations. Thus, the input by the system may deliver new inputs for exploration and learning. 

## 6. Discussion of Specific HCI Challenges in the Front End of Medicine

**Figure 7:**
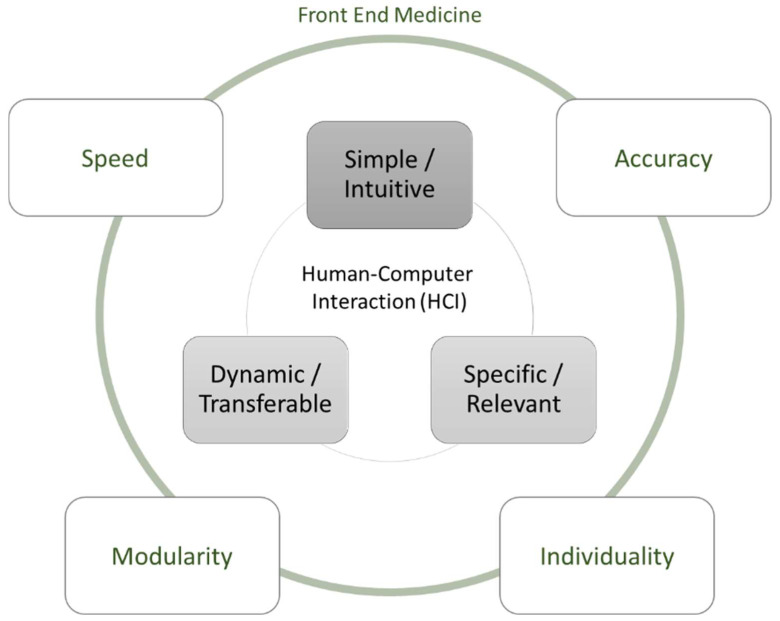
Concept draft of the front end of medicine HCI requirements.

The implementation of digital applications as outlined by the three case studies highlights the relevance of detailed process and requirements analyses, especially in health care contexts. Specific requirements toward HCI concepts arise from the complex environment of health care (e.g., its multitude of stakeholders) and a dedicated process complexity due to highly specialized work tasks and a high division of labor for a series of actors and organizations in this sector. Figure 7 addresses the four specific requirements within front-end medicine processes in detail. 

*Accuracy*: For the Münster use case, accuracy plays a central role given that information regarding severely injured persons is transported digitally to the medical center. Therefore, data input and specific information sequences are of high relevance. This is addressed in the digital application, where specific fields for medical data are included. For the second case from Göttingen, the central information required is compressed into codes. Accuracy is the essential criterion when transmitting this information since individual letters and numbers determine the diagnosis and treatment of the patient. Therefore, the automatic extraction is additionally validated by the medical expert if the algorithm is below the set threshold of accuracy. For the third case from Göttingen, medical insights such as the relevance of certain factors for treatment recommendations were at the center of the user’s interest in the developed tool. Accordingly, the accuracy of the information is of major importance. Although this information does not automatically trigger actions, it is meant to enrich and support expert discussions. 

In sum, all cases share the need for high levels of accuracy. This insight underscores the importance of medical information in all phases of the medical front end. While the cases reveal differences in how this information is processed (e.g., automatically vs. manually) or the purpose of their use (e.g., treatment vs. administrative decisions), the accuracy of specific patient-related information is essential. 

*Speed*: Within use case 1 in Münster, the time needed for patient transport to the medical center location is crucial for treatment and survival probability. Therefore, information and decision speed for emergency personnel is one of the elements of HCI concepts for the emergency medicine sector. For use case 2, the automation of information extraction is intended to save time and manual working steps for the medical personnel. The information is transferred immediately to all necessary systems and is in this way available to the medical staff in the respective environments. In this case, speed represents a central HCI element. In use case 3, speed is an important issue. While the tool is not used for each and every decision in everyday practice, it needs to deliver results almost immediately after the surgeons request it. The tight schedule of the surgeons demands that calculation times are minimized and the tool is always ready. The DoL architecture allows for increased speed. 

In sum, the cases reveal that speed in HCI is of particular relevance to accelerate the front end of medical processes. On the one hand, medical conditions can create temporal pressures (e.g., emergency cases). On the other hand, the temporal resources of associated experts (e.g., surgeons) are scarce. The time needed for HCI, either in entering, retrieving, or transferring medical information, must be minimized. 

*Modularity*: Within the first application case in Münster, a multitude of systems are included in emergency medicine processes and therefore modular setups are required. For example, this is implemented with picture and voice recording options in the developed application for EMS teams, thereby enabling individual development and innovation for digital EMS features. The second case deals with bridging the modularity of the systems used so that information is automatically transferred to systems. The developed tool also consists of a modular structure, which essentially comprises two components: feature extraction and expert interaction. While the tool developed in the reported third case from Göttingen does not encompass multiple systems, its DoL architecture and the differentiation of Experts and CEs are inherently modular. 

In sum, the cases illustrate the importance of modularity in the technical architectures underlying HCI at the medical front end. Although variations exist in the embedding of the respective digital applications in further systems, this insight underscores the complexity of medical processes and medical decisions. 

*Individuality*: As different user groups are involved in the first case for data processing during emergency medicine activities (i.e., EMS doctors, hospital doctors, EMS assistants, or drivers/pilots), individual handling and adjustment options are included in the implemented HCI concept. For example, emergency doctors can include hospital ratings and notes for future decision situations where the digital application can report on past experiences and rating data. Although the second use case from Göttingen focuses on standardized automation, the EIM requires individual feedback from physicians. Thus, transparency for the physicians is given to promote the improvement of automatic extraction. The interface is designed to show the information in a clear, traceable manner to avoid the impression of a black-box algorithm. While the system in the third case is developed for a particular hospital department, the surgeons involved varied in their experience, fields of expertise, and digital nativeness. Therefore, it was of utmost importance to display the information neutrally and clearly so that the experts could absorb them in an unbiased manner for their particular purpose. Furthermore, it was crucial to not create technological barriers hindering the accessibility of the information. 

In sum, the three application cases show that individuality is a particularly relevant aspect for HCI at the medical front end. The cases illustrate that a multitude of different stakeholder groups involved in the respective processes interact with the digital applications. In addition, individuals within a particular stakeholder group vary substantially in their traits and capabilities. 

The described case studies and the derived concept drafts as exploratory research approaches are limited in their validity due to the small empirical sample base [24]. This limitation warrants further research with additional cases and quantitative confirmatory studies initiated by the presented discussion draft for a health care HCI concept. Altogether, the three use cases have shown in great practical detail that the four highlighted requirements for HCI concepts in processes regarding the front end of medicine are crucial for the efficient and effective implementation of digital solutions. 

On a strategic level, results and insights from this HCI requirements draft can be connected to two major discourse areas. First, the extension of requirement items leads to differing objectives with competing interests in HCI design. For example, increasing accuracy in data collection will work against speed and simplicity. Therefore, a core requirement for digital systems in front-end medicine sectors is to balance these conflicting objectives. To illustrate, the emergency medicine case study from Münster was confronted with the requirement of collecting patient age as an important input factor for later treatment decisions in severe trauma cases. However, implementing a text field or pulldown list for specific age data input (e.g., 68 years) would have had a negative impact on speed and simplicity for users. Therefore, it was decided that only three age groups (<15, 15–65, >65) can be ticked, making the interaction very fast and simple. This approach combines medically relevant information with the requirements of speed and simple data processing interaction for emergency medical personnel. Second, typically for front-end medicine systems, different knowledge bases are addressed and required. Besides medical knowledge, areas such as administration, organization, logistics, or other relevant topics and persons are required and integrated. These two specific framework characteristics have to be considered for successful HCI design within the front end of medicine.

## 7. Outlook

This paper provided detailed insights into the specific requirements and restrictions of digital applications in the front-end areas of medical processes, that is, before patient treatment starts [80,81,82,83,84]. These include digital support for EMS, data extraction in inpatient processes, and cancer diagnosis processes. As also represented by these cases, typical hospital processes during a pandemic can be improved and optimized during pandemic events like COVID-19—as shown for example for training HCI applications in [85].

An overview was presented regarding common elements, identifying especially the necessary extraction of specific medical requirements in all three exemplary cases with elaborate instruments like process analyses, interviews, or other instruments. Moreover, the extraordinary relevance of HCI concepts and their adaption to specific health care management requirements were highlighted. An adapted HCI concept was also drafted for further evaluation and discussion. Further empirical research is highly warranted as these success factors for digital application projects in health care are the basis for effective digital transformations with a high level of improvement expectations attached.

## Figures and Tables

**Table 1:** Accuracy of Feature Extraction.

Feature	Manual Coding	Automated Transmission
Neo. therapy	99.19%	98.78%
T	99.59%	97.97%
N	99.59%	98.78%
X1/2	98.38%	97.57%
M	99.59%	99.59%
L	100%	100%
V	100%	100%
R	100%	98.78%
Complete data	97.57%	93.52%

**Table 2:** Overall Correctness of Feature Extraction.

Extraction Method	True	False	Ambiguous	Total
Manual Coding	241 (97.57%)	6	-	247
Automated Transmission	233 (93.52%)	5	9	247

**Table 3:** Most Important Attributes and Scores for Each Prediction Task (Tofangchi et al. 2017).

Most Important Patient Attributes for Predictions					
Decision Category	Attribute	Score	Decision Category	Attribute	Score
Therapy Intention	Lymph node-(N)-classification	0.825	Systemic Chemotherapy	Tumor-(T)-classification	0.692
	Tumor-(T)-classification	0.438		Diagnosis admission status	0.505
	Metastasis-(M)-classification	0.332		Clinical state	0.441
	Grading of squamous cell carcinoma	0.202		Tumor number	0.357
	Clinical state	0.129		Tumor type	0.337
	Height	0.0714		Reason for visit	0.278
	Tumor number	0.0698		Sex	0.274
	Familial tumor predisposition	0.0648		Height	0.211
	Reason for visit	0.0568		Familial tumor predisposition	0.185
Operation	Metastasis-(M)-classification	0.276		Amount of alcohol	0.169
	Grading of squamous cell carcinoma	0.146	Other Therapy	Clinical state	0.906
	Lymph node-(N)-classification	0.145		Reason for visit	0.358
	Tumor type	0.0592		Duration of precancerous lesions	0.109
	Tumor-(T)-classification	0.0494		Tumor type	0.0811
Radiation	Tumor-(T)-classification	0.366		Tumor-(T)-classification	0.0550
	Lymph node-(N)-classification	0.322	Overall	Tumor-(T)-classification	0.323
	Inflammatory response	0.0789		Clinical state	0.299
	Degree of dysplasia	0.0533		Lymph node-(N)-classification	0.273
	Existing precancerous lesions	0.0430		Reason for visit	0.140
Local Chemotherapy	Heavy heart disease	0.0790		Metastasis-(M)-classification	0.120
	Grading of squamous cell carcinoma	0.0682			
	Lymph node-(N)-classification	0.0675			
	Restricted lung functions	0.0554			
	Existing precancerous lesions	0.0454			

**Table 4:** Scores of Therapy Predictors.

	Therapy Intention	Operation	Radiation	Local Chemo-therapy	Systemic Chemo-Therapy	Other Therapy	Overall
Random Guesser	0.120	0.610	0.315	0.00752	0.234	0.0153	0.218
Prior Classifier	0	0.961	0	0.286	0	**0.571**	0.308
Prior Classifier DoL	0	0.961	0	0.286	0	**0.571**	0.308
LASSO	0	**0.966**	0.48	0.213	0.240	0	0.320
LASSO DoL	0.434	0.958	0.430	0.259	0.259	0.286	0.439
Logistic Regression	0.452	0.958	0.458	0.285	0.285	**0.571**	0.500
Logistic Regression DoL	**0.649**	0.957	**0.528**	**0.496**	**0.561**	**0.571**	**0.620**
LPM	0.336	0.945	0.457	0.127	0.127	0.146	0.358
LPM DoL	0.526	0.946	0.454	0.139	0.139	0.146	0.392
Decision Tree	0.308	0.932	0.355	0.240	0.22	0.223	0.382
Decision Tree DoL	0.455	0.937	0.448	0.304	0.258	0.242	0.442
SVM	0.260	0.957	0.397	0.286	0.300	**0.571**	0.465
SVM DoL	0.41	0.959	0.513	0.476	0.517	**0.571**	0.577
DNN	0.0317	0.851	0.104	0.143	0.0395	**0.571**	0.316

Note: F_1_ scores of different classifiers for the prediction of therapy intentions and each treatment type. The overall score is the average of the scores for all prediction tasks, weighted by the number of samples in the respective test sets. The best score is in bold text for each prediction task [72]. Additional information about the approaches listed in Table 4 [72]: 1. Random guesser: guesses the class of a data element at random. 2. Prior classifier: always predicts the class has the most occurrences in the data set. 3. LASSO classifier: LASSO regressor extended to classification problems. - predicts class 0, if the regressor predicts a value smaller than 0.5. - predicts class 1 otherwise. 4. Logistic regression [73]. 5. Linear Probability Model (LPM) [74]. 6. Decision tree: trained with the CART algorithm [75]. 7. Soft-margin support vector machine (SVM) [76]. 8. Deep neural network (DNN) [77] with four hidden layers.
